# A Novel Three-Dimensional Glioma Blood-Brain Barrier Model for High-Throughput Testing of Tumoricidal Capability

**DOI:** 10.3389/fonc.2019.00351

**Published:** 2019-05-03

**Authors:** Hilary Sherman, Ann E. Rossi

**Affiliations:** Life Sciences Division, Corning Incorporated, Kennebunk, ME, United States

**Keywords:** blood-brain barrier, cancer, glioma, spheroid, three-dimensional cell culture, Transwell

## Abstract

The blood-brain barrier (BBB) limits passage of substances between general circulation and the brain extracellular fluid, maintaining homeostasis in neural tissues and providing a defense against potential toxins. However, the protection provided by the BBB often prevents conventional chemotherapeutics from reaching brain tumors which makes brain cancers one of the most difficult cancers to treat (1). Traditionally, high-throughput testing of compound permeability through the BBB *in vitro* has been limited to assay of radio- or fluorophore-labeled compounds as they pass a cell monolayer growing on a permeable support system. Unfortunately, the labels themselves may negatively impact the assay, and the ability to determine resulting tumor cytotoxicity must be studied independently. The present study demonstrates proof-of-concept of a three-dimensional (3D) model to study label-free BBB transport as well as the resulting brain tumor cytotoxicity by combining two commercially available products: Corning® HTS Transwell®-96 tissue culture system and Corning 96-well spheroid microplates. Transwells are permeable support systems commonly used for drug transport and migration/invasion studies (2, 3). Corning spheroid microplates are cell culture microplates with round well-bottom geometry coated with Corning Ultra-low Attachment surface, enabling the formation of a single multicellular tumor spheroid centered in each well in a highly reproducible manner. By replacing the standard flat-bottom Transwell receiver plate with a Corning spheroid microplate, the resulting system—which can be tailored to any number of cell types and screening applications—enables a more comprehensive assay to study drug transport across the BBB and the resulting 3D glioma spheroid toxicity in an easy-to-use 3D high-throughput assay.

## Introduction

The blood-brain barrier (BBB) is a complex microvascular network consisting primarily of astrocytes, pericytes, and endothelial cells that functions as a gatekeeper between the general circulation and the brain extracellular fluid (1, 4). Though water, oxygen, and other hydrophilic substances can cross the barrier freely, only the smallest of lipophilic molecules can pass and passage of any substance is tightly controlled. Selective permeability of the BBB is maintained by: (1) the physical barrier of tight junctions between endothelial cells lining cerebral microvessels; (2) uptake and efflux transporters that control transcellular movement of ions, nutrients, toxins, drugs, and xenobiotics; and (3) cellular enzyme systems that metabolize biologically active molecules such as catecholamines and acetylcholine (1, 4). Control of the passage of substances across the BBB is essential for both maintaining homeostasis and protecting the central nervous system (CNS) from toxins and pathogens (4). Yet, it is precisely this natural barrier function that often prevents therapeutics from reaching the brain and spinal fluid, which makes many neurological disorders notoriously difficult to treat. For example, because many cancer drugs cannot adequately penetrate the BBB and are thus ineffective, the BBB has been cited to contribute to the difficulty in brain tumor treatment, adding to the poor prognosis following diagnosis (1, 2). Identifying novel mechanisms to bypass barrier function will result in more effective treatment modalities for both CNS cancers and other neurological disorders.

Current research in this area of drug discovery employs a variety of *in vitro* models that are each effective in recapitulating specific properties of the BBB, such as tight-junction formation and transporter function (4, 5). The BBB has traditionally been studied by culture of primary or immortalized brain endothelial cell lines as mono-cultures or as co-cultures with astrocytes and pericytes (5). To test compound permeability through *in vitro* BBBs, researchers assay radio- or fluorophore-labeled compounds as they pass cell monolayers growing upon the permeable support system. In addition, unlabeled compounds can be studied via liquid chromatography–mass spectrometry (LCMS) or other analytical chemistry techniques. While traditional methods have reasonable throughput for screening potential CNS-targeted therapeutics, they are limited to study of only BBB permeability or effects on the barrier itself and do not test functionality of compounds once they pass the BBB. Action on the brain and other CNS tissues by positive hits identified in these types of screens requires independent validation. Accordingly, the authors sought to develop a more comprehensive model, eliminating the need for individual assays. Like the novel 3D immune oncology model developed by the authors (6), the current work demonstrates the feasibility of combining two commercially available products—Corning® HTS Transwell®-96 tissue culture system and Corning 96-well spheroid microplates—to generate a 3D model to study label-free BBB transport as well as the resulting brain tumor cytotoxicity. By combining a simple, traditional permeable support BBB model with 3D glioma spheroids cultured in the spheroid microplate, study of the BBB permeability of chemotherapeutics and resulting tumor cytotoxicity is possible in a single, easy-to-use 3D high-throughput assay.

## Materials and Methods

### Transwell Blood-Brain Barrier

MDCKII/MDR1 cells were obtained from Dr. Piet Borst (Netherlands Cancer Institute, Amsterdam, Netherlands) and seeded into the apical chamber of HTS 96-well Transwells (Corning Cat. No. 7369) at 1 × 10^5^ cells/cm^2^ in 100 μL of Dulbecco's Modification of Eagle's Medium (DMEM; Corning Cat. No.10-013-CM) supplemented with 10% fetal bovine serum (FBS; Corning Cat. No. 35-010-CV). Media (25 mL) was added to the basal reservoir plate. Cells were cultured for 5 days with a full media exchange in both chambers 24 h prior to assay. Monolayer integrity and P-glycoprotein (P-gp) pump function were assessed via lucifer yellow (LY; Sigma-Aldrich Cat. No. L0144) permeability and rhodamine 123 efflux (Rh123; Sigma-Aldrich Cat. No. R8004), respectively. Briefly, 100 μL of 60 μM LY in Hank's Balanced Saline Solution (HBSS; Corning Cat. No. 21-021-CM) containing 1% DMSO was added to the apical chamber of the Transwells as well as 150 μL of HBSS containing 1% DMSO in the basolateral compartment of the receiver plate. The concentration of LY that reached the basolateral compartment was measured as fluorescence after 60 min via a PerkinElmer EnVision Multimode Plate Reader and reported as nm/s. In a similar manner, Rh123 fluorescence was measured in the apical and basolateral compartment following application of 100 μL in the apical compartment and 150 μL in the basolateral compartment of different wells. Rh123 was added at a concentration of 50 μM diluted in HBSS containing 1% DMSO. The receiving chamber (apical or basolateral) was filled with HBSS containing 1% DMSO of either 100 or 150 μL, respectively. Rh123 transfer to the opposite compartment was similarly read on the PerkinElmer EnVision Multimode Plate Reader and reported as nm/s or apparent permeability (Papp).

MDCKII/MDR1 monolayers were immunostained with Alexa Fluor® 488-conjugated ZO-1 (Thermo Fisher Cat. No. 339188) and Alexa Fluor® 488-conjugated Occludin (Thermo Fisher Cat. No. 331588) per manufacturer's protocol to confirm the presence of tight junction proteins. Nuclei were counterstained with 1.8 μM Hoechst 34580 (Thermo Fisher Cat. No. H21486). Stained monolayers were imaged directly on intact Transwell inserts with a Thermo Scientific CellInsight CX7. Images were processed with ImageJ using the smoothing filter and rolling ball background subtraction (250 pixel radius).

### 3D Glioma Spheroids

LN-229 cells (ATCC® Cat. No. CRL-2611™) to be used for the glioblastoma model were routinely cultured in DMEM containing 10% FBS. Cells were harvested with Accutase® cell detachment solution (Corning Cat. No. 25-058-CI), seeded into the Corning 96-well spheroid microplates (Corning Cat. No. 4520) at 1 × 10^3^ cells in 50 μL per well, and incubated for 24 h prior to assay to form 3D spheroids. For initial drug testing, spheroids were exposed to varying concentrations of cisplatin (Sigma-Aldrich Cat. No. 1134357) and piperlongumine (Sigma-Aldrich Cat. No. 528124) by adding 50 μL of drug or vehicle control and culturing for 48 h. Cisplatin was reconstituted in Dulbecco's Phosphate-Buffered Saline (DPBS; Corning Cat. No. 21-031-CM) and piperlongumine in DMSO. Both were diluted to final concentrations with media. After drug exposure, 100 μL/well of CellTiter- Glo® 3D (Promega Cat. No. G9683) was added to assess cell viability, and the spheroid microplates were processed according to manufacturer's instructions. Luminescence was detected using a PerkinElmer EnVision Multimode Plate Reader.

### BBB-Glioma Spheroid Model

**Figure 1:**
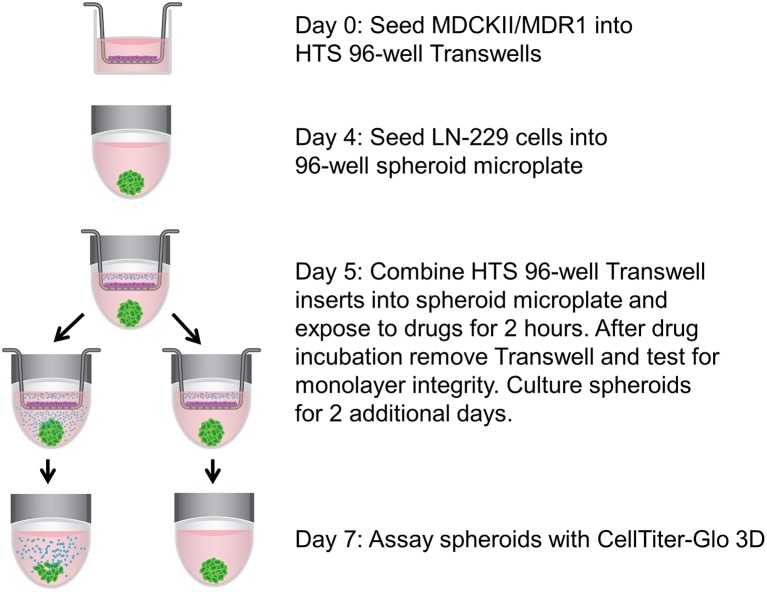
Schematic of assay setup. MDCKII/MDR1 cells were cultured as a monolayer in HTS 96-well Transwell inserts. While the *in vitro* BBB was forming, LN-229 cells were seeded in a spheroid microplate to generate glioma spheroids, which then became the receiver microplate for the Transwell inserts. Upon addition of cytotoxic drugs to the apical chamber of the inserts, the components were incubated together temporarily to allow for drug diffusion across the BBB. Spheroids were cultured for an additional 2 days without inserts, then assayed for cell viability with CellTiter-Glo 3D.

MDCKII/MDR1 monolayers were cultured on Transwells for 5 days. Immediately prior to combining with the spheroid microplate, media was removed from both basal and apical chambers of the Transwells, and the apical chamber media was replaced with 100 μL of 250 μM cisplatin in media, piperlongumine in media, or media containing vehicle controls. Inserts with and without MDCKII/MDR1 monolayers were then combined with spheroid microplates, which contained 24-h-old LN-229 spheroids in 200 μL of media per well, and incubated for 2 h at 37°C. A schematic of the combined setup is shown in Figure 1. After 2 h of co-incubation, inserts were removed, tested for LY permeability to monitor barrier integrity, and discarded. LN-229 spheroids were returned to the incubator for two additional days and then assessed for viability as described above with the following exception: media was removed and replaced with a 200 μL of CellTiter-Glo 3D diluted 1:1 with media.

To investigate the utility of the BBB-glioma model for screening, BBB monolayers and LN-229 spheroids were set up as described. After 5 days of BBB formation, media was removed from both basal and apical chambers of the Transwells, and the apical chamber media was replaced with 50 μL of 1 mM compound from the Tocriscreen Kinase Inhibitor Toolbox (Tocris Bioscience Cat. No. 3514) in media or media containing vehicle. Inserts were then combined with 24-h-old LN-229 spheroids for 2 h at 37°C. After 2 h of co-incubation, inserts were removed to test for barrier integrity, and LN-229 spheroids were cultured and assayed as described.

### Statistics

All data is graphed as mean ± SD. Statistical significance was determined by two-tailed *t*-test using GraphPad Prism.

## Results and Discussion

### Blood-Brain Barrier Model

**Figure 2:**
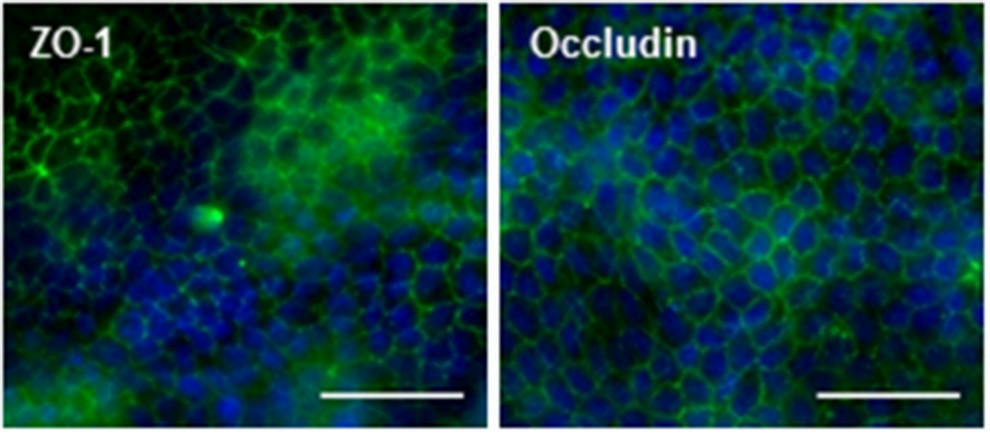
Presence of tight junction proteins in MDCKII/MDR1 monolayer. Representative confocal images of ZO-1 **(left)** and occludin **(right)** immunofluorescence (green) showing characteristic tight junction localization between cells of the monolayer. Nuclei are counterstained with Hoechst (blue). Images were acquired with a 40X objective on the Thermo Scientific CX7 CellInsight and processed with ImageJ using the smoothing filter and rolling ball background subtraction (250 pixel radius). Scale bars = 50 μm.

**Figure 3:**
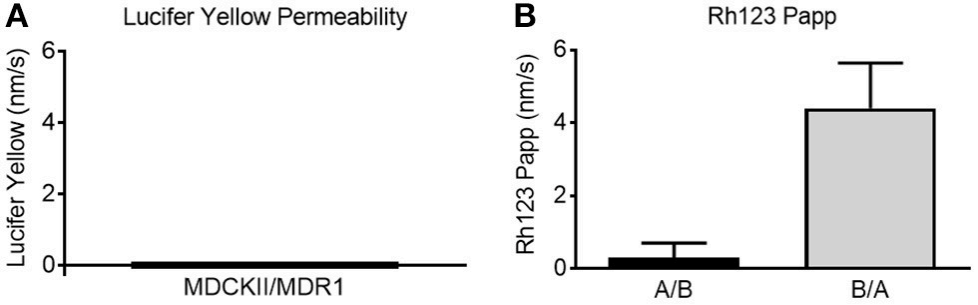
MDCKII/MDR1 cell monolayers form functional barriers. **(A)** Lucifer yellow permeability was measured at 0 nm/s. Low lucifer yellow permeability (<20 nm/s) indicates a tight monolayer (9). **(B)** Rh123 apparent permeability (Papp) indicated stronger efflux in the basolateral-to-apical (B/A) compared to the apical-to-basolateral direction (A/B). Efflux ratio was > 2 nm/s, indicative of functional P-gp pumps (10). Mean ± SD. *N* = 72 for three independent tests.

Historically, MDCKII/MDR1 cells have been used to create *in vitro* models to test for BBB permeability (7, 8). The MDCK model as a surrogate BBB demonstrates functional efflux (i.e., P-gp pump activity) characteristic of the BBB, has been shown to distinguish between CNS-penetrant and non-penetrant compounds, and has been validated by *in situ* rat brain perfusion studies (8). To demonstrate the effectiveness of our MDCKII/MDR1 BBB model, we assessed formation of tight junctions, monolayer integrity, and P-gp pump function. The formation of tight junctions was verified by immunofluorescence staining of the tight junction-associated proteins ZO-1 and occludin. Confocal microscopy images clearly show positive staining for both proteins (Figure 2). The fluorescence pattern encircling each cell —visible even with light scattering caused by imaging the monolayer through the Transwell membrane —is consistent with localization of tight junctions between cells of the monolayer. The next step in validating the Transwell BBB was to assess the tightness of the monolayer. By measuring leakage of LY from the apical compartment of the Transwell insert to the basolateral compartment over the course of 1 h, the integrity of the monolayer can be quantified. Cell monolayers with LY permeability values < 20 nm/s are considered intact (9). In this study, the LY permeability value was 0 nm/s, consistent with a continuous monolayer (Figure 3A). Additionally, to function as an *in vitro* BBB the MDCKII/MDR1 would require functional P-gp pumps, the major efflux transporter with broad substrate specificity. P-gp pump activity is commonly measured by comparing the basolateral-to-apical efflux and apical-to-basolateral efflux of Rh123, a known substrate of the P-gp pump. Figure 3B shows higher efflux of Rh123 basolateral-to-apical direction, i.e., active transport Rh123 against its concentration gradient. Comparison of the basolateral-to-apical efflux and apical-to-basolateral efflux yields an efflux ratio of 4.4 ± 1.2 nm/s, indicative of a functioning P-gp pump (10). Though the MDCKII/MDR1 BBB model utilized in this study is best suited to screening P-gp substrates, the concept of a BBB cultured on a permeable membrane can easily be adapted to other cell types which higher expression of additional efflux transporters such as members of the multidrug resistance protein (MRP) family and breast cancer resistance protein (BCRP).

### Spheroid Toxicity

**Figure 4:**
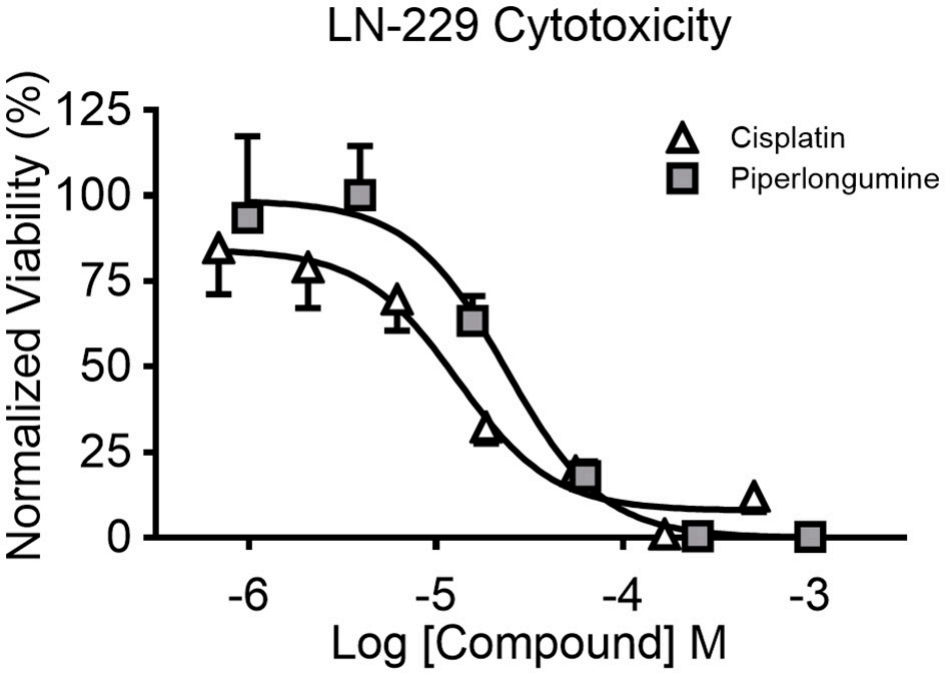
Concentration-dependent cytotoxicity of LN-229 spheroids. LN-229 viability after 48 h of direct exposure to varying concentrations of cisplatin (white triangles) and piperlongumine (gray squares). Normalized viability was calculated by comparing luminescent signal in the presence of drug to that of buffer plus vehicle controls. Mean ± SD. *N* = 12 wells per concentration from two independent studies.

The Transwell BBB model was paired with 3D glioblastoma spheroids to create a more physiological *in vitro* system to investigate the effect of chemotherapeutic agents on brain tumors. Simply, the HTS Transwell system was directly coupled with the spheroid microplate containing pre-formed LN-229 glioma spheroids (Figure 1). LN-229 cells were chosen for this study because they are derived from a human glioblastoma and are commonly used for GBM research (11, 12). LN-229 readily form spheroids and were a starting point to demonstrate the feasibility of the model. Though they do not recapitulate the heterogeneity of GBM tumors, they are a simple, inexpensive model for high-throughput screening applications. Certainly, LN-229 could easily be replaced with other GBM cell types or co-culture for target-molecule validation or for specific research applications. To demonstrate the utility of the BBB model, the effects of two known cytotoxic drugs, cisplatin and piperlongumine, were tested first directly on LN-229 spheroids then with addition of the BBB. Cisplatin is known to induce cytotoxicity in cancer cells but is incapable of penetrating the BBB at high enough levels to be effective (13). On the other hand, piperlongumine has not only been shown to induce cancer-specific cytotoxicity but is known to cross the BBB at high levels (14–16). A concentration-dependent response was achieved when both compounds were added directly to LN-229 spheroids at varying concentrations, with IC_50_ values of 13 and 24 μM for cisplatin and piperlongumine, respectively (Figure 4).

**Figure 5:**
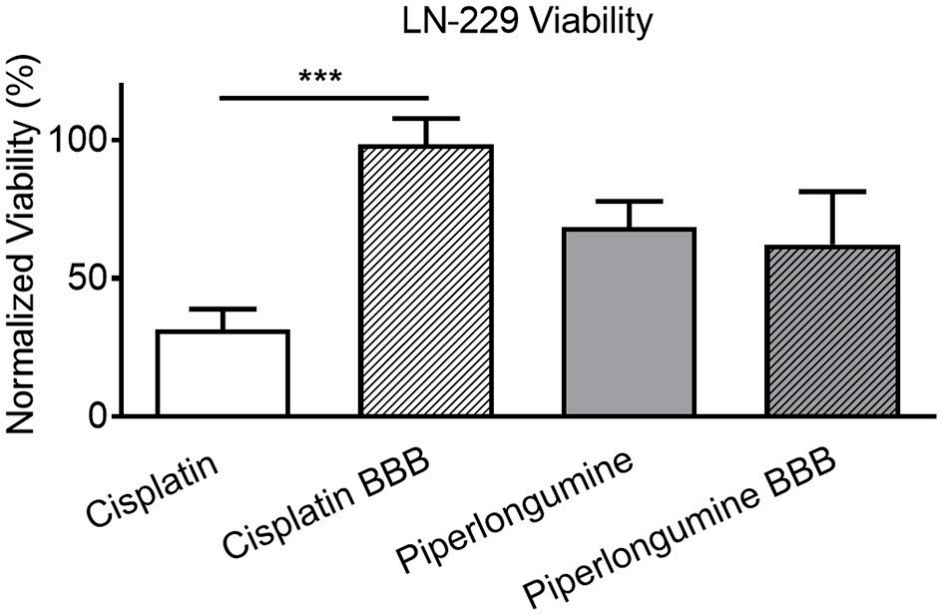
Model demonstrates *in vivo*-like cytotoxic response. LN-229 viability 48 h after a 2h exposure to 250 μM cisplatin or piperlongumine. Percent viability was calculated by comparing luminescent signal in the presence of drug to that of buffer plus vehicle controls. Two-tailed *t*-test indicated a statistically significant (*p* < 0.0001) difference between cisplatin-induced cytotoxicity with and without a BBB. Mean ± SD. *N* = 30 from three independent studies. ^***^ denotes level of statistical significance (*p* < 0.0001).

**Figure 6:**
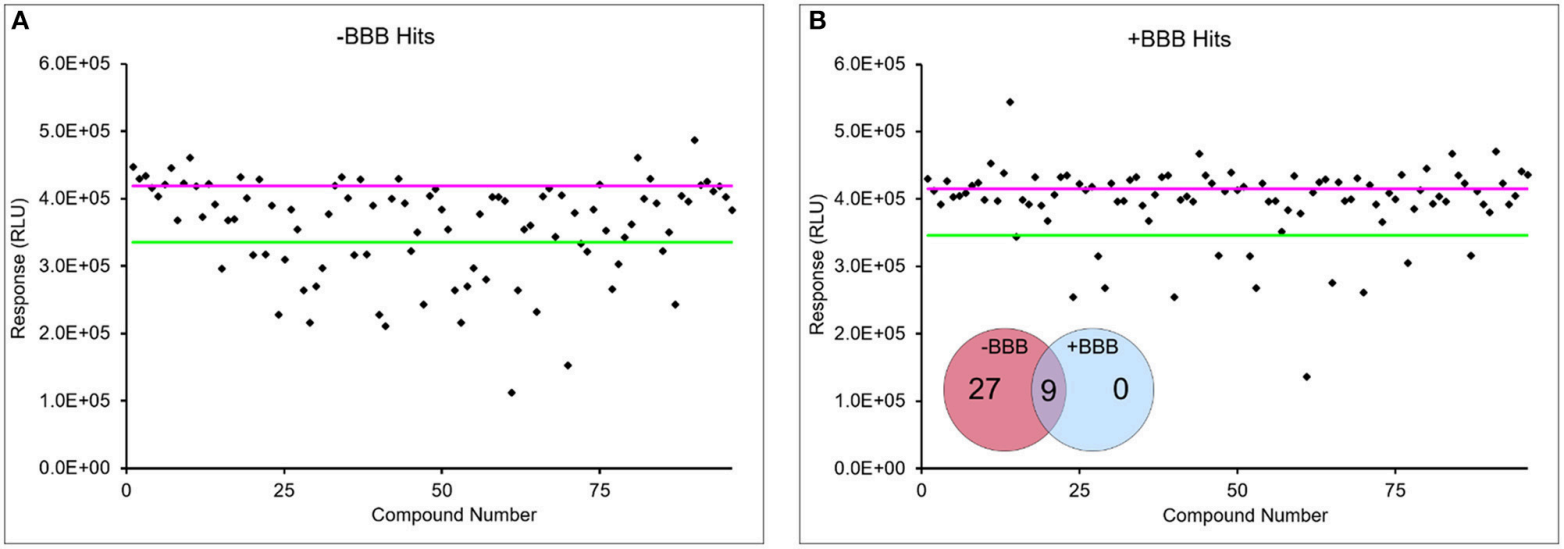
BBB narrows list of cytotoxic hits in small library screen. Representative LN-229 viability data with **(B)** and without BBB model **(A)** from screen of 80-drug library. Pink line is average buffer response and green line represents 3 σ below buffer response. Data points below green line were considered hits. Hits were considered if they were 3 σ below buffer response in at least two out of three independent screens. A fraction of hits in the absence of BBB were identified in the presence of BBB from three screens (inset).

For the combined model, drug concentrations higher than the IC_50_ values were added to the apical chamber to ensure sufficient concentrations of drugs would pass the BBB in the limited incubation time with the spheroid microplate. Importantly, the combination of the HTS 96-well Transwells with the LN-229 spheroids recapitulated the *in vivo* behavior of the two drugs (Figure 5). Piperlongumine induced cytotoxicity regardless of the presence of a BBB. In contrast, there was a statistically significant reduction in cytotoxic effect observed with cisplatin when the BBB was present. The BBB model was further tested with a screen of a small library of kinase inhibitors to determine if cytotoxicity of the glioma spheroids was dependent upon BBB permeability. Kinase inhibition is currently a target for several different cancer therapeutics, and the Tocris library provided a small curated group of compounds to test in this proof-of-principle study. Figure 6 shows a representative screen of the drug library and buffer controls performed with and without a BBB on the HTS 96-well Transwell inserts. A hit was identified as a compound with a relative luminescent (RLU) value of <3 σ of the buffer response. More cytotoxic hits were identified in the absence of the BBB: 27 in total. In three independent screens, only nine of those hits (Figure 6, inset) were also positive with the addition of the Transwell BBB. Importantly, co-culture of the spheroids with BBB narrowed the hit list. This subset represents true positives in the assay.

## Conclusions

Certainly, traditional *in vitro* BBB models cultured in two-dimensions (2D) upon permeable membranes have proven effective to study various aspects of BBB physiology and pathophysiology, including barrier permeability and transport mechanisms and have been key in cell-based screening assays for CNS-targeted therapeutics (17). Newer models of the BBB employ 3D cell culture to model the complex cell-cell interactions intrinsic to the *in vivo* BBB and represent an improvement upon monolayer BBB cultures. For example, Cho et al. presented a multicellular spheroid model generated by co-culture of primary human astrocytes and human brain vascular pericytes with two different human brain EC types on a low-attachment surface. The cells self-assemble into spheroids with the surface of the spheroid demonstrating barrier function, including tight junction formation and efflux transporter activity. Specific substrate transport was monitored with fluorescently-labeled angiopep-2 substrate (18). However, as singular models, both these 2D and 3D models are limited to study of only the barrier itself and do not test functionality of compounds once they pass the BBB. Separate studies are required to investigate compound effects on the CNS independently from BBB transport. Likewise, any compounds found to be active on the CNS must be counterscreened for the ability to pass the BBB. A more comprehensive model would eliminate the need for individual assays. To this end, the authors developed a model to identify therapeutic compounds that can both permeate the BBB and induce cytotoxicity of tumor spheroids. The BBB represents a significant obstacle to drug delivery to the CNS and thus, contributes to the dearth of effective therapies for a variety of diseases of the CNS. Among the many diseases, brain tumors have a particularly poor prognosis. For these reasons, a model system to investigate BBB transport and glioma spheroid cytotoxicity were a logical starting place for a more comprehensive BBB model. In this initial feasibility study, the MDCKII/MDR1 and LN-229 cell lines were chosen as basic models of the BBB and GBM, respectively.

The authors took additional cues from their previously published immune-oncology model (6), which combined Corning 96-well spheroid microplates and HTS 96-well Transwell inserts. The BBB has historically been modeled *in vitro* on permeable supports with a variety of cell types (5). As proof-of-principle, MDCKII/MDR1 cell monolayers were cultured on the HTS 96-well Transwell inserts. The Transwell BBB was coupled with LN-229 spheroids—a simple 3D GBM model—cultured in the 96-well spheroid microplate, as tumor cells cultured as 3D spheroids have been shown to more closely replicate tumor microenvironments (6, 19–21). Importantly, the *in vitro* BBB modeled in the current study was shown to possess typical BBB barrier function and indeed controlled the passage of known chemotherapeutics, cisplatin, and piperlongumine, with the expected cytotoxicity on the glioblastoma spheroids. Further, the sample screen identified gefitinib as a true positive hit, exhibiting cytotoxicity on the glioblastoma spheroid in both the absence and presence of the BBB, consistent with literature reports (22). The combined model was successful in narrowing the hits of cytotoxic compounds from a small, representative compound library, which shows it can eliminate need for a separate BBB permeability counterscreen and clearly demonstrates the utility of the model. For future validation, this proof-of-concept study can be extended to include more clinically relevant drugs, such as temozolomide.

Moreover, the model presented here can be easily adjusted to incorporate other cell types and expanded to additional applications. The BBB could be generated using mono or co-cultures including primary endothelial cells/astrocytes/pericytes, immortalized human endothelial cells, or stem cell-derived neurons, as has been reviewed in the literature (5), to more closely mimic the complexity of the BBB. Similarly, the “target” cell cultures in the spheroid microplate can be tailored to model the heterogeneity of glioblastomas and other CNS tumors, such as patient-derived GBM neurospheres. In addition, the combined model can be expanded to several different screening applications. For example, Sirenko et al. generated human induced pluripotent stem cell (hiPSC) 3D neural cultures to investigate neurotoxic effects of a small library of compounds that included pharmaceutical drugs (23). Addition of the Transwell BBB to such screening models would parse out toxic compounds that healthy CNS tissue is normally protected from by an intact BBB. By extension, the combined Transwell-spheroid culture system could be utilized in more complex research studies of neuroimmunological and neuroinflammatory mechanisms. Redondo-Castro and colleagues examined cytokine signaling by placing human MSC spheroids in permeable supports and assessing the effects of their secretome on BV2 microglial cells in culture (24). Anti-inflammatory markers were identified in MSC spheroid-conditioned media, especially if the spheroids were primed with interleukin-1 (IL-1). Yet, the authors did not observe anti-inflammatory effects on LPS-treated BV2 cells in co-culture (24). It is conceivable that the context-poor environment of the 2D BV2 cultures was not primed to trigger response to the spheroid secretome. The same model could be recreated in a single culture system by culturing the microglial cells as 3D structures in the spheroid microplate. Further, addition of an *in vitro* BBB would enable simultaneous study of known MSC migration across the BBB (24).

Regardless of the cell type(s) chosen, the system described here represents a more comprehensive, more *in vivo*-like model with which researchers can test the ability for a compound, cell or cell product to pass the BBB while simultaneously looking at the impact on a 3D structure, in a single easy-to-use assay.

Ultimately, these types of advanced *in vitro* models that more closely mirror conditions *in vivo* will accelerate drug discovery and development of CNS-targeted therapeutics.

## Author Contributions

HS designed the study, performed all experiments, and analyzed data. HS and AR prepared the manuscript for publication. Both authors have read and approved the final manuscript.

### Conflict of Interest Statement

HS and AR are employed by Corning Incorporated.
